# Toxicity and toxicokinetic assessment of an anti-tubercular drug pretomanid in cynomolgus monkeys

**DOI:** 10.1016/j.toxrep.2022.04.021

**Published:** 2022-04-22

**Authors:** Rebecca Bruning-Barry, Jeffrey L. Ambroso, John Dillberger, Tian J. Yang

**Affiliations:** a Global Alliance for TB Drug Development, New York, NY, USA; b RTI International, Research Triangle Park, NC, USA; c J. Dillberger LLC, Nashville, IN, USA

**Keywords:** PA-824, Toxicity, Monkeys, Nonclinical safety assessment

## Abstract

Pretomanid is a nitroimidazooxazine antimycobacterial drug that was approved in more than 10 countries as part of a three-drug, all oral regimen, consisting of bedaquiline, pretomanid, and linezolid (BPaL) for 6-months treatment of adults with pulmonary extensively drug-resistant tuberculosis (XDR-TB) or with complicated forms of multidrug-resistant tuberculosis (MDR-TB). The toxicological profile of pretomanid was thoroughly evaluated in repeat-dose oral toxicity studies up to 39 weeks long in cynomolgus monkeys. Exposures up to 10-fold higher than in humans at the approved pretomanid dose (200 mg) were achieved in acute studies allowing for characterization of dose-limiting toxicity. Target organs and processes identified in acute and chronic toxicity studies included QT prolongation, nervous system effects, and liver effects (minimal hepatocellular hypertrophy without elevations in liver enzymes). In a 13-week study, no cataracts were present at the end of dosing, but 2 of 12 monkeys had cataracts at the end of a 13-week recovery period. No cataracts related to pretomanid administration were observed in subsequent 13-week or 39-week studies. No male reproductive toxicity was observed in these studies. No-observed-adverse-effect levels (NOAELs) were identified in all studies. Exposures at the NOAELs equaled, or exceeded, human exposure at the approved pretomanid dose with the exception of female monkeys in a 39-week chronic toxicity study. These data support the use of pretomanid as part of the 6-month BPaL regimen for treating XDR-TB and MDR-TB.

## 1 Introduction

Pretomanid (also known as PA-824) is a newly approved nitroimidazooxazine antimycobacterial drug with a complex mechanism of action that has not been fully elucidated but involves inhibition of cell wall mycolic acid biosynthesis under replicating (aerobic) conditions and respiratory poisoning through generation of reactive nitrogen species, including nitric oxide (NO), under non-replicating (anaerobic) conditions [1], [2], [3]. In August 2019, pretomanid was the second antibacterial drug product approved by the United States Food and Drug Administration (FDA) under the Limited Population Pathway for Antibacterial and Antifungal Drugs (LPAD pathway). It has since been approved by the European Medicines Agency, India, Democratic Republic of the Congo, Georgia, Kazakhstan, Moldova, Mozambique, South Africa, South Korea, Tajikistan, Turkmenistan, Ukraine, Uzbekistan, Zimbabwe and has been prequalified by the World Health Organization (WHO) Prequalification of Medicines Programme. Pretomanid is indicated for the treatment of adults with complicated forms of multi-drug resistant tuberculosis (MDR-TB, ie., resistant to isoniazid and rifampicin) or with pulmonary extensively drug-resistant tuberculosis (XDR-TB [pre-2021 revised WHO definition [4]]) which is MDR-TB with additional resistance to fluroquinolones and second-line injectables as part of a three-drug, all oral, 6-month regimen containing pretomanid, bedaquiline and linezolid (BPaL).

Tuberculosis (TB) treatment is complicated, requiring a combination of drugs that are selected based on known or expected *Mycobacterium tuberculosis (*M.tb) sensitivity to medications, and can last from six months to longer than two years. Drug-sensitive TB is treated with a standard 6-month course of 4 first-line antimicrobial drugs (isoniazid, rifampicin, pyrazinamide, and ethambutol) that have been around for decades. However, drug-resistant TB affects approximately 500,000 people each year and is a major contributor to antimicrobial resistance worldwide [5]. Pretomanid was approved for complicated forms of MDR-TB, which includes cases where M.tb does not respond to treatment or where treatment is discontinued because of toxicity. XDR-TB is a category of MDR-TB that is additionally resistant to any fluoroquinolone and to at least one of three injectable second-line drugs (amikacin, kanamycin, or capreomycin) [4], [6]. Treatment of XDR-TB or complicated MDR-TB has historically been lengthy and complex, requiring a combination of as many as seven antibiotics, some involving daily injections, for 18-months or longer [6], [7]. The recent approvals of bedaquiline, delamanid, and pretomanid over the past decade, along with the repurposing of linezolid as an antituberculosis drug, have been major steps forward for the management of drug-resistant TB, enabling all-oral treatment regimens, including some of significantly shorter duration and improved treatment success [8]. Bedaquiline and delamanid were approved as add-ons to current, complex MDR-TB regimens, whereas pretomanid was approved in the specific context of a novel 3-drug, all oral regimen (BPaL), exemplifying a new approach to TB treatment development [8]. The BPaL regimen successfully treated 90% of XDR-TB and complicated MDR-TB after 6 months of treatment, which is comparable to outcomes with the standard of care for drug-sensitive TB [7].

To support the clinical development of pretomanid and its market approval as part of the BPaL regimen in multiple countries, the toxicological profile of pretomanid was evaluated as a single drug in repeat-dose oral toxicity studies up to 26 weeks long in rats and 39 weeks long in cynomolgus monkeys. These studies were part of a comprehensive nonclinical safety evaluation program conducted in accordance with applicable GLP regulations and regulatory guidance documents. This manuscript describes the toxicity and toxicokinetic profiles of pretomanid in monkeys given day oral doses for 1, 2, 13, and 39 weeks.

## 2 Materials and methods

### 2.1 Test article and formulations

**Fig. 1:**
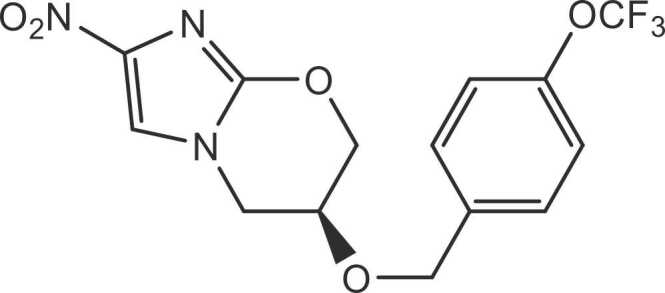
Chemical structure of pretomanid.

Pretomanid, (6 *S*)− 2-nitro-6-{[4-(trifluoromethoxy)benzyl]oxy}− 6,7-dihydro-5*H*-imidazo[2,1-*b*] [1,3] oxazine, was synthesized by Cambridge Major Laboratories Inc (≥99.8% purity by HPLC area%) and Metrics (≥99.8% purity by HPLC area%). The molecular structure of pretomanid is shown in Fig. 1. The formulations used in the monkey toxicity studies were suspensions either in 0.4% or 0.5% carboxymethylcellulose (designated as CMC, suppliers Sigma Aldrich [Batch No. 046K0050] or Spectrum Chemical Mfg. Corp [Lot No. 1CA0148, 2CE0360, 2CI0310, TC1083]), with or without 0.1% Tween 80, or in 10% hydroxypropyl-β-cyclodextrin (suppliers Research Diagnostics Inc. [Lot No. 82003/H3U171P] or Carghill Inc. [Lot No. H3N188P]) and 10% lecithin (supplier Natermann Phospholipid Gmbh [Batch 20441]) [w/v] in purified water (designated as CM2).

### 2.2 **Animals**

Male and female cynomolgus macaques (*Macaca fascicularis*) were obtained from Covance Research Products, Inc. (United States) for the 1-, 2-, and 13-week general toxicity studies; SNBL USA (United States) for a 13-week investigative toxicity study focused on reproductive and ocular effects, and Huazheng Laboratory Animal Breeding Center (China) for the 39-week general toxicity study. Monkeys were allowed to acclimate to the laboratory conditions for at least 3 weeks before being enrolled in a study. Approval from the Institutional Animal Care and Use Committees (IACUC) at the contract research organizations (Covance Laboratories Inc, MPI Research, Inc, SRI International, and SNBL USA) were obtained before using these animals in the nonclinical safety studies. All animal experiments were conducted in compliance with the Animal Welfare Act, the Guide for the Care and Use of Laboratory Animals, and the Office of Laboratory Animal Welfare.

Monkeys were selected as the nonrodent species for toxicology testing of pretomanid due to rapid clearance and poor absorption in dogs, resulting in very low systemic exposures regardless of the formulation and route of administration tested. Although the pharmacokinetic profile of pretomanid was evaluated in dogs, no toxicology studies were done in this species.

At the initiation of dosing in the 1-, 2- and 13-week studies, monkeys were approximately 17 months to 3 years old, and their body weights were between 1.5 and 2.5 kg. At the initiation of dosing in the 13-week investigative study and 39-week study, monkeys were sexually mature, 4.5–7 years old, and weighed 3–8.2 kg. Monkeys were housed individually in stainless steel cages under standard conditions of temperature, humidity, ventilation, and illumination. Tap water was provided ad libitum, and monkey laboratory diet was offered once or twice per day.

### 2.3 Experimental designs

An overview of the experimental designs for the monkey toxicity studies are presented in Table 1. The 1- and 2-week studies were conducted at Covance Laboratories Inc (Vienna, VA)[9], [10], the 13-week general toxicity study was conducted at MPI Research Inc (Mattawan, MI)[11], the 13-week investigative toxicity study was conducted at SNBL USA (Everett, WA)[12], and the 39-week general toxicity study was conducted at SRI International (Menlo Park, CA)[13]. The studies were performed in compliance with Good Laboratory Practice (GLP) regulations with the exception of the 1-week study, which was exploratory and conducted under the contract research organization’s (CRO’s) standard operating procedures. No impactful GLP exceptions or study deviations were noted.

**Table 1:** Overview of Experimental Designs for Monkey Toxicity Studies with Pretomanid.

Study description	Dose route	Vehicle	Dose levels (Dose volume)	Total duration of dosing^a^ (No. of monkeys per group)	Duration of treatment-free recovery period (No. of monkeys/group)
1-Week study (Non-GLP)	Nasogastric gavage	CM2	100 or 250 mg/kg/day (5 mL/kg)	1-Week (1 M + 1 F)	–
2-Week study (GLP)	Nasogastric gavage	CM2	0, 50, 150, 450, or 1000 mg/kg/day(10 mL/kg)	2-Weeks (5 M + 5 F for vehicle and HD groups and 3 M + 3 F for LD and MD groups)	2-Weeks (2 M + 2 F for vehicle and HD groups)
13-Week study (GLP)	Oral gavage	0.4% CMC	0, 50, 150, or 450/300^b^ mg/kg/day (5 mL/kg)	13-Weeks^b^(6 M + 6 F for vehicle and HD groups and 4 M + 4 F for LD and MD groups)	13-Weeks (2 M + 2 F for vehicle and HD groups)
13-Week study – male reproductive function and lenticular lesions (GLP)	Nasogastric gavage	0.5% CMC	0, 50, 150, or 300 mg/kg/day (5 mL/kg)	13-Weeks^c^(8 M for vehicle and HD groups and 5 M for LD and MD groups)	20-Weeks (3 M from LD and MD groups^d^ and 4 M for vehicle and HD groups)
39-Week study (GLP)	Oral or nasogastric gavage	0.5% CMC + 0.1% Tween 80	0, 25, 50, or 100 mg/kg/day (5 mL/kg)	39-Weeks (5 M + 5 F for vehicle, 4 M + 4 F for LD and MD, and 7 M + 7 F for HD)	13-Weeks (2 M + 2 F for vehicle and HD groups)

Abbreviations: No. = Number, M = Males, F = Females, - = Not applicable; HD = High dose; LD = Low-dose; MD = Mid dose., Vehicles: CM2 = 10% hydroxypropyl-β-cyclodextrin and 10% lecithin [w/v] in purified water; 0.4% CMC = aqueous sodium carboxymethylcellulose, medium viscosity in deionized water; 0.5% CMC + 0.1% Tween 80 = 0.5% (w/v) aqueous sodium carboxymethylcellulose, medium viscosity in 0.1% (v/v) polysorbate 80 in sterile water; 0.5% CMC = 0.5% (w/v) carboxymethylcellulose medium viscosity in sterile water for injection.

a The number of monkeys listed in the total duration of dosing column includes monkeys that remained on the study during the treatment-free period.

b Dosing at 450 mg/kg/day stopped on Day 27 and resumed at 300 mg/kg/day on Day 37 for all animals except one, which dosing resumed on Day 40.

c Pretomanid dosing was suspended or terminated early due to treatment-emergent toxicity at 150 or 300 mg/kg/day with males administered test article for 84 or 47–84 days, respectively.

d Following treatment termination, the protocol was amended to allow 3 animals from Groups 2 and 3 to recover for 20 weeks.

Doses were administered once daily by oral or nasogastric gavage at a dosing volume of 5 or 10 mL/kg (Table 1). Except for the 1-week study, a vehicle control group was included for comparison and post-treatment assessment periods, referred to as treatment-free or recovery periods, were included to evaluate reversibility, progression, or delayed toxicity. Toxicokinetic evaluations were included in all studies to evaluate systemic exposure to pretomanid.

### 2.4 Data collection systems

The CROs utilized a number of GLP compliant data collection systems to monitor the animal room temperature and humidity, facility storage conditions where test material was stored, document transfer of study materials, perform statistical analyses, archive study materials, and to collect inlife and clinical and anatomic pathology data. These records are archived at the CROs. Major computer systems used to collect, analyze, and report the inlife data included, but were not limited to, Provantis™ v6.5 or v8.6, Randomization and Data Extension System (RADES), Automatic Form and Label Generation System (AFLGS), Test Article Logging In, Storage, and Management computer system (TALISMAN), Dispense™ v5.0, Data Extraction Linked to Versatile Electronic Reporting system (DELIVER), Data Acquisition Program (DAP), Labcat version 4.42, SAS v8.2, Stata 13 SE, MPI Reporting System v7.0, ChemLog database, REES environmental monitoring, Computer Assisted Sperm Analysis (CASA-IVOS), and eNotes.

The 1-week study evaluated the toxicity and maximum tolerated dose (MTD) of pretomanid in pairs of monkeys (1/sex) given pretomanid at 100 or 250 mg/kg/day and then euthanized and necropsied after the last dose.

The 2-week study evaluated the toxicity and toxicokinetics of pretomanid in groups of monkeys given vehicle (CM2) or pretomanid at 50, 150, 450, or 1000 mg/kg/day. The control and high-dose groups had 10 monkeys (5/sex), and the other groups had 6 monkeys (3/sex). Monkeys were euthanized and necropsied after the last dose (3/sex/group) or after a 16-day recovery period (2/sex in control and high-dose groups).

The 13-week general toxicity study evaluated the toxicity and toxicokinetics of pretomanid in groups of monkeys given vehicle (0.4% CMC) or pretomanid at 50, 150, or 450/300 mg/kg/day. The control and high-dose groups had 12 monkeys (6/sex), and the other groups had 8 monkeys (4/sex). Monkeys were euthanized and necropsied after the last dose (4/sex/group) or after a 13-week recovery period (2/sex in control and high-dose groups). Due to toxicity, dosing at 450 mg/kg/day stopped on Day 27 and resumed at 300 mg/kg/day on Day 37 for all animals except one female, which resumed dosing on Day 40.

The 13-week investigative toxicity study evaluated the effects of pretomanid on the reproductive system and lens of the eye in groups of sexually mature male monkeys given vehicle (0.5% CMC) or pretomanid at 50, 150, or 300 mg/kg/day. The control and high-dose groups had 8 monkeys, and the other groups had 5 monkeys. Monkeys were euthanized and necropsied after the last dose (5 each in the control and high-dose groups and 2 each in the other groups) or after a 20-week recovery period (3/group). At the start of treatment, the monkeys weighed 4.5–7.1 kg and were 4.7–6.9 years old. They were included in the study if their baseline semenology data and male reproductive endocrine status were within the normal range for sexually mature monkeys and if they had no lens lesions. The semenology and reproductive criteria for study selection were the confirmed presence of descended testicles of at least 15 mL in combined volume, adequate sperm motility (>70%) and sperm concentration (>200 ×10^6^/mL) in samples collected by electro-ejaculation, and serum testosterone concentration > 5.0 ng/mL. The control and 50 mg/kg monkeys were dosed for 13 weeks (91 days). Due to treatment-emergent toxicity, monkeys in the 150 and 300 mg/kg/day groups were dosed daily for 13 weeks with a 7-day holiday (from Days 36–42), resulting in a total of 84 dose administrations. Additionally, dose administration was discontinued for four animals in the 300 mg/kg/day group on Day 56, 59, or 75; total dose administrations for this group ranged from 47 to 84 days. Dose holidays or dose termination were required because the animals were not eating well and had lost substantial body weight.

The 39-week general toxicity study evaluated the toxicity and toxicokinetics of pretomanid in groups of monkeys given vehicle (0.5% CMC) or pretomanid at 25, 50, or 100 mg/kg/day. The control group had 6 monkeys (3/sex), the low- and mid-dose groups had 8 monkeys (4/sex), and the high-dose group had 10 monkeys (5/sex). Monkeys were euthanized and necropsied after the last dose (1/sex in the control group, 4/sex in the low- and mid-dose groups, and 3/sex in the high-dose group) or after a 13-week recovery period (2/sex in control and high-dose groups).

### 2.5 In-life observations and measurements

Monkeys were observed for clinical signs of toxicity (including qualitative effects on food consumption) and effects on body weight and clinical pathology parameters. In the 2-, 13-, and 39-week general toxicity studies, electrocardiograms (ECG) were evaluated. In the 2-, 13-, and 39-week general toxicity studies and the 13-week investigative toxicity study, ophthalmic examinations were conducted.

### 2.6 Mortality, clinical observations, and body weights

Animals were checked for mortality, abnormalities, and signs of pain and distress twice daily (a.m. and p.m.). Detailed clinical observations were performed at least once prior to the dosing phase, at least once weekly during the dosing phase, and on the day of scheduled sacrifice. The detailed clinical observations included but were not limited to, evaluation of the skin, fur, eyes, ears, nose, oral cavity, thorax, abdomen, external genitalia, limbs and feet, respiratory and circulatory effects, autonomic effects such as salivation, and nervous system effects including tremors, convulsions, reactivity to handling, and abnormal behavior. Daily cageside observations were performed prior to dosing and at approximately 1 and 4 h postdose. Additional clinical findings were recorded when they were observed, and in the case of the 13-week general toxicology study, additional observations were scheduled postdose during week 10 once an hour for five consecutive hours to monitor for signs of convulsions and/or seizures. During the recovery phase, cageside clinical observations were performed once daily. Food consumption was assessed once daily during dosing and recovery phases. Body weights were measured at least once prior to the dosing phase, on the first day of treatment, and at least once weekly during the treatment and recovery phases.

### 2.7 Clinical pathology

Blood for clinical pathology (standard hematology, clinical chemistry, and coagulation [most studies] panels) was collected prior to the dosing phase, before terminal sacrifices, and near the end of the recovery phase. In the 13-week investigative study, coagulation parameters were not assessed, and additional serum hormone concentrations were measured (testosterone [ELISA kit, IBL America], inhibin-B, luteinizing hormone [LH], and follicle stimulating hormone [FSH]). Inhibin-B, LH, and FSH were measured by radioimmunoassay and/or ELISA at the Oregon National Primate Research Center. Interim clinical pathology evaluations were conducted during week 4 in the 13- and 39-week studies and week 26 in the 39-week study. Urine was also collected for urinalysis around the same time as blood samples in the general toxicity studies.

### 2.8 ECG exams.

A board-certified veterinary cardiologist evaluated ECGs in the 2-week study, monkeys were anesthetized with ketamine for ECGs. The ECGs were recorded prior to the dosing phase for all monkeys, during the estimated T_max_ on Days 2 and 15 between 2 and 4 h postdose for the control, 50, and 150 mg/kg/day groups, and 5–7 h postdose for the 450 and 1000 mg/kg/day groups, and all remaining animals at the end of recovery. ECGs were assessed for qualitative and quantitative changes in this study.

In the 13-week general toxicity study, non-sedated monkeys had ECGs recorded twice prior to the dosing phase, predose and 5–6 h postdose on Days 7 and 90 of the dosing phase, and prior to recovery necropsy. Standard 10-lead ECGs were recorded at 50 mm/sec and evaluated qualitatively for abnormal configurations and rhythms.

In the 39-week study, anesthetized monkeys had ECGs recorded prior to the dosing phase, during weeks 13, 26, and 39 of the dosing phase, and near the end of the recovery phase. Standard 10-lead ECGs were recorded at 50 mm/sec and 10 mm/mV sensitivity and were assessed for qualitative and quantitative changes.

### 2.9Ophthalmic exams

A board-certified veterinary ophthalmologist examined eyes using an indirect ophthalmoscope and a slit lamp biomicroscope. The ophthalmic exams were conducted while the monkeys were anesthetized with ketamine. Prior to examination, a mydriatic agent was used in both eyes.

In the 2- and 13-week general toxicity studies, eyes were examined prior to the dosing phase and before the terminal and recovery necropsies. In the 39-week study, eyes were examined prior to the dosing phase, in weeks 7, 13, 20, 26, 33, and 39 of the dosing phase, and twice during recovery (7 and 12 weeks into recovery). In the 13-week investigative toxicity study, eyes were examined twice prior to the dosing phase, in weeks 3, 8, and 13 of the dosing phase, and in weeks 3, 7, 12, and 17 of the recovery phase.

### 2.10 Necropsy and pathology

Full necropsies were performed on the day after the last dose was administered and after the recovery phases per the study designs listed in Table 1. Animals were euthanized using IACUC- and veterinary-approved procedures which included sedation with ketamine, followed by an intravenous overdose of sodium pentobarbital and exsanguination. During the necropsy, animals’ musculoskeletal system, external carcass and body orifices were examined for abnormalities. The abdominal, thoracic, and cranial cavities were examined for abnormalities and the organs removed, examined, and where required, weighed, and/or placed in fixative. All designated tissues were fixed in 10% neutral buffered formalin except for the eyes and testes which were fixed using a modified Davidson’s fixative. Tissue samples were embedded in paraffin, sectioned, and stained with hematoxylin and eosin for examination by light microscopy. For all studies except the 13-week investigative study, standard tissue lists were examined microscopically in accordance with the ICH and FDA guidelines. In the 13-week investigative study, the standard list of tissues was collected, but only testes, epididymides, eyes, pituitary, prostate gland, and seminal vesicles were examined microscopically.

### 2.11 Bioanalytical and toxicokinetic (TK) assessment

Blood samples (~1.0-mL) were collected via femoral vein or other suitable peripheral vessel into K_3_EDTA tubes after the first dose and prior to terminal sacrifice, except in the 7-day study where no samples were taken after the first dose. Seven to ten sampling timepoints were selected to profile the toxicokinetics, including some or all of the following timepoints: predose and 0.5, 1, 2, 4, 6, 8, 12 or 16, and 24 h postdose. Additional blood was collected for interim TK profiles on Day 7 in the 13-week general toxicity study, on Days 35 and 50 in the 13-week investigative study, and on Days 15 and 90 in the 39-week study.

Samples were centrifuged to obtain plasma and stored frozen (−60 to −80 °C) until analyzed for pretomanid concentrations by high-performance liquid chromatography (HPLC) with tandem mass spectrometric (MS/MS) detection (method validated at Covance Laboratories Inc., Madison, WI).

Individual animal pretomanid plasma concentration-time profiles were subjected to noncompartmental pharmacokinetic analysis using WinNonlin Phoenix (version 6.3) or Professional Edition (Pharsight Corporation, version 3.3 or 5.2). Concentration values below the lower limit of quantitation (<10.0 ng/mL) were treated as zeros or excluded (39-week study only) for descriptive statistics and TK analysis. The following parameters and constants were determined: maximal plasma concentration (C_max_), time to maximum plasma concentration (T_max_), area under the plasma concentration-time curve to twenty-four hours postdose (AUC_0–24 h_), and terminal elimination half-life (t_1/2_).

### 2.12 Statistical analysis

Descriptive statistics including mean and standard deviation were performed for selected parameters. If sample sizes were three or greater a Bartlett or Levene’s test were used to assess homogeneity of group variances for pre-specified study endpoints and collection intervals. If the Bartlett or Levene’s test were not significant (p ≥ 0.01 [13-week general tox study only] or p ≥ 0.05), pooled estimates of the variance were computed from a one-way analysis of variance (ANOVA) and Dunnett’s tests were utilized for the comparison of treatment groups with the control group. If Levene’s test were significant (p < 0.01 [13-week tox study only] or <0.05), comparisons with the control group were conducting using Welch’s t-test. Bonferroni corrections were applied to t-tests in the 2-week and 13-week studies. For the 13-week investigative study, if the Bartlett’s test was significant, the data were analyzed by Kruskal-Wallis H-test, and depending on if the sample sizes were equal or unequal, the Miller’s procedure or Dunn’s modification were used to compare the treatment groups back to the control group, respectively. With the exception of descriptive statistics, statistical analysis was not conducted for the 7-day study because it was an exploratory study and did not contain a control group.

### 2.13 Margin of safety

The recommended dose of pretomanid in the BPaL regimen is 200 mg/day, which produced average steady-state C_max_ and AUC_0–24 h_ values in humans of 2.9 µg/mL and 50.9 h* µg/mL, respectively (unpublished data, Nix-TB Trial, ClinicalTrials.gov Identifier NCT02333799). The latter exposure was sex combined exposures from n = 27 participants from the Nix Phase 3 clinical trial after 16-weeks administration of bedaquiline (400 mg once daily for 2 weeks followed by 200 mg three times a week, tablet), pretomanid (200 mg once daily, tablet), and linezolid (1200 mg once daily, tablet) (ClinicalTrials.gov Identifier: NCT02333799, [7]). These pretomanid exposures from the Nix-TB trial were compared to exposures in monkeys at the NOAEL to calculate the margin of safety.

## 3 Results

Doses evaluated in repeat-dose toxicity studies in monkeys ranged from 25 to 1000 mg/kg/day. Longer treatment durations correlated with lower no-observed-adverse-effect levels (NOAELs, Table 3). Key findings from these studies are described below and included in Table 2, Table 3, Table 4, and 5.

**Table 2:** Noteworthy Findings in the Pivotal Monkey Toxicity Studies with Pretomanid.

Duration of Dosing	2-Weeks				13-Weeks				39-Weeks			
Dose (mg/kg/day)	450		1000		150		450/300^a^		50		100	
Sex and No. Terminal/ Recovery Animals^b^	M 3/2	F 3/2	M 3/0	F 3/0	M4/0	F 4/0	M 4/2	F 4/2	M 4/0	F 4/0	M 5/2	F 5/2
Mortality^c^	–	–	–	–	–	–	–	–	1^d^	–	–	–
Body Weight^a^	0.85x*	0.89x*	0.80x*	0.78x*	0.85x	0.91x	0.78x*	0.74x*	0.94x	0.78x	0.77x*	0.78x
CV Effects												
Heart Rate Change (Δbpm)^e^	-30 *	-21 *	-40 *	-27 *	–	–	–	–	–	–	–	–
Prolonged QT Interval (≥250msec)^c^	2	3	3	3	–	–	–	–	–	–	–	–
Nervous System Effects												
Convulsions^c^	–	–	–	1	–	–	2	–	–	–	1	–
Tremors, limb^c^	–	–	1	–	–	–	–	–	–	–	–	–
Ataxia^c^	–	–	–	2	–	–	1	2	–	–	–	–
Cataracts												
Posterior cortical^f^	–	–	–	–	–	–	0/1	0/1	1	–	–	–
Liver Effects												
Organ weight^a^	–	–	–	–	1.4x*	1.4x*	1.5x*	1.7x*	1.2x	1.1x	1.4x*	1.1x
Hepatocyte hypertrophy^c^												
Minimal	–	–	–	–	–	–	–	4	–	–	–	–

Abbreviations: CV = Cardiovascular; M = Males; F = Females; Δbpm = change in beats per minute *Denotes statistical difference vs. control (p < 0.05)

a Multiples of body or organ weight (% body weight) vs. control at the end of the dosing period. Statistics are performed on actual data and not multiples.

b Number of animals is presented in the row as sex: number of animals per group.

c Data is presented as the number of animals effected. QT interval prolongation was in excess of normal range defined as ≥ 250 msec.

d The animal died during surgery to remove a mass in the intrapleural space and the cause of death was not attributed to treatment with pretomanid.

e Group mean change in heart rate vs. predose at the end of the dosing period (Day 15)

f No ocular abnormalities occurred during the dosing phase of the 13-week study, but cortical axial posterior cataracts were observed in 1 M and 1 F at the end of the recovery period. In the 39-week study, one male administered 50 mg/kg/day had 3 punctate posterior cortical cataracts in both eyes at the end of the dosing phase but were considered likely related to faulty lens epithelial adhesion and were not attributed to treatment with pretomanid.

**Table 3:** Repeat-dose NOAELs and Associated Steady-state Plasma Exposures to Pretomanid.

Study Duration	Sex	NOAEL (mg/kg)	C_max_ (µg/mL)		AUC_0–24 h_ (µg*h/mL)	
2 weeks						
Male		150		15.7		194
Female		150		15.9		195
13 weeks						
Male		50		7.2		78
Female		50		8.9		96
39 weeks						
Male		50		5.2		66
Female		25		2.2		20
Human^a^		200 mg (fixed)		2.9		50.9

Abbreviations: AUC_0–24 h_ = area under the concentration-time curve from time 0–24 h post-dose; C_max_ = maximum concentration; F = female; M = male; NOAEL = no-observed-adverse-effect-level

a Sex combined exposures from n = 27 participants from the Nix Phase 3 clinical trial (ClinTrials.gov Identifier: NCT02333799, [Conradie et al., 2020 [7]]) after 16-weeks administration of bedaquiline (400 mg once daily for 2 weeks followed by 200 mg three times a week, tablet), pretomanid (200 mg once daily, tablet), and linezolid arm (1200 mg once daily, tablet).

**Table 4:** Toxicokinetic Data from the First and/or Last Dose of the 1-Week, 2-Week, and 13-Week Toxicity Studies with Pretomanid.

Study	Dose	Day	Sex	T_max_ (h)	C_max_ (µg/mL)	t_1/2_ (h)	AUC_0__-24__h_ (µg*h/mL)
1-Week	100	7	M	4	13.0	3.1	137
			F	2	10.1	3.1	90
	250	7	M	8	19.1	8.6	320
			F	4	14.2	4.0	194
2-Week	50	1	M	2	6.0	3.5	52
			F	5	7.2	4.2	80
		14	M	3	7.1	3.4	54
			F	5	8.5	3.7	88
	150	1	M	5	12.4	6.3	177
			F	6	18.1	5.1	258
		14	M	3	15.7	5.1	194
			F	4	15.9	4.0	195
	450	1	M	8	20.7	6.6	353
			F	10	25.3	6.9	439
		14	M	6	24.1	9.3	420
			F	8	22.8	9.5	405
	1000	1	M	11	28.6	NA	559
			F	11	24.5	NA	492
		14	M	4	28.2	NA	540
			F	4	25.5	NA	470
13-Week	50	1	M	4	4.4	3.3	44
			F	4	5.7	4.4	69
		90	M	6	7.2	3.9	78
			F	5	8.9	3.9	96
	150	1	M	5	10.9	4.2	112
			F	4	14.2	5.1	169
		90	M	6	14.8	6.6	195
			F	7	17.3	3.9	262
	450/300^a^	1	M	8	33.1	10.6	566
			F	7	37.3	11.4	638
		90	M	6	18.5	5.0	297
			F	5	19.0	8.7	292

Abbreviations: M = Male, F = Female; NA = Not applicable; AUC0–24 h = area under the concentration-time curve from time 0–24 h post-dose; Cmax = maximum concentration; Tmax = Time to maximum observed concentration; t1/2 = Elimination half-life

a Dosing was discontinued at 450 mg/kg/day on Day 27. Dosing resumed at 300 mg/kg/day on Day 37, except for one female, which resumed dosing at 300 mg/kg/day on Day 40.

**Table 5:** Toxicokinetic Data from the First and Last Dose of the 13-Week Investigative Toxicity Study and the 39-Week Toxicity Study with Pretomanid.

Study	Dose	Day	Sex	T_max_ (h)	C_max_ (µg/mL)	t_1/2_ (h)	AUC_0–24 h_ (µg*h/mL)
13-Week Investigative Study	50	1	M	6	4.1	4.9	54
		87	M	4	5.8	7.3	70
	150	1	M	13	9.9	NA	186
		87	M	6	12.2	4.4	196
	300	1	M	13	12.9	NA	247
		87	M	9	16.2	NA	267
39-Week	25	1	M	4	6.3	5.6	47
			F	3	2.7	2.5	23
		260	M	4	4.8	4.5	43
			F	3	2.2	4.3	20
	50	1	M	4	5.5	4.4	53
			F	6	4.9	2.9	50
		260	M	4	5.2	4.9	55
			F	3	4.6	4.4	45
	100	1	M	6	8.1	3.1	99
			F	5	7.4	6.0	97
		260	M	5	6.8	5.4	89
			F	5	10.0	4.8	124

Abbreviations: M = Male, F = Female; NA = Not applicable; AUC_0–24 h_ = area under the concentration-time curve from time 0–24 h post-dose; C_max_ = maximum concentration; T_max_ = Time to maximum observed concentration; t_1/2_ = Elimination half-life

### 3.1 1-week MTD study

Monkeys tolerated daily doses of pretomanid well at 100 or 250 mg/kg. The only drug-related findings were minor fecal abnormalities and low food consumption on Days 2 through 7; therefore, the 7-day MTD was 250 mg/kg/day. After the last dose at the 7-day MTD, mean sex-combined C_max_ and AUC_0–24 h_ values were 16.7 µg/mL and 257 µg*h/mL, respectively.

### 3.2 2-week general toxicity study

Monkeys tolerated daily doses of pretomanid at 50, 150, 450, or 1000 mg/kg, albeit with adverse findings at ≥ 450 mg/kg/day. Key toxicity findings are presented in Table 2.

Clinical signs indicative of nervous system effects included ataxic behavior, convulsions followed by lateral recumbency and ataxia, or front limb tremors, which were observed in a few animals at 1000 mg/kg/day (Day 14 sex combined C_max_ and AUC_0–24 h_ = 26.9 µg/mL and 505 µg·h/mL, respectively, Table 4).

Mean body weights were significantly decreased compared with control at 450 mg/kg/day beginning on Day 11 for males and Day 13 for females, and for both males and females administered 1000 mg/kg/day on Day 11. A cumulative mean body weight loss of 0.1–0.2 kg from predose was observed for males at 450 mg/kg/day and both sexes at 1000 mg/kg/day and correlated with thin appearance for main study animals during necropsy and increased incidence of low and/or no food consumption in these animals during the dosing phase. Monkeys administered 150 mg/kg/day also were noted to have increased incidence of low food consumption during the dosing phase. One male administered 450 mg/kg/day had reduced food consumption for seven days into the 16-day recovery period while food consumption stabilized in the remaining animals. During recovery, mean body weight gains of the 450 mg/kg/day monkeys exceeded that of the control monkeys by 0.2 kg, indicating this change was reversible.

Dose- and time-related decreases in heart rate and increases in QT interval were observed on Days 2 and 15, mainly in groups administered ≥ 450 mg/kg/day (Day 14 sex combined C_max_ and AUC_0–24 h_ at 450 mg/kg/day = 23.5 µg/mL and 413 µg·h/mL, respectively, Table 4). ECG changes returned to baseline values by the end of the recovery phase for animals administered 450 mg/kg/day. Because of the use of anesthesia, a limited number of ECG tracings, an inability to compare animals with similar heart rates, and the lack of a validated heart rate correction formula for QT interval in this species, definitive conclusions on pretomanid’s effect on QT interval, independent of heart rate, could not be made.

All ophthalmic examinations were normal.

Notable clinical pathology findings included moderate reductions in reticulocyte counts at 450 and 1000 mg/kg/day (−49% and −67% compared with controls, respectively).

There were no macroscopic observations at necropsy with findings limited to decrease in absolute and relative thymic weights for monkeys administered ≥ 450 mg/kg/day. Decreased thymic weights correlated with minimal to moderate lymphoid depletion in the thymus of monkeys administered ≥ 450 mg/kg/day. Thymic findings were resolved in the 450 mg/kg/day recovery monkeys except for one male. There were no other pretomanid-related microscopic findings in any other tissues evaluated including the liver and reproductive organs.

There were no marked sex differences in exposure or accumulation of pretomanid after repeat administration. Exposure to pretomanid increased with increasing dose and was dose proportional between 50 and 150 mg/kg/day. A further increase in dose resulted in less than proportional increase in exposure (Table 4).

The 2-week NOAEL was considered to be 150 mg/kg/day, based on the occurrence of effects on the nervous or cardiovascular systems at the 450 mg/kg/day dose level. After the last dose at the NOAEL, the sex-combined C_max_ and AUC_0–24 h_ were 15.8 µg/mL and 195 µg*h/mL, respectively.

### 3.3 13-week general toxicity study

Daily oral doses of pretomanid were well tolerated at 50 mg/kg/day, tolerated but with adverse findings at 150 mg/kg/day, and not tolerated at 450 mg/kg/day. At 450 mg/kg/day dosing was stopped on Day 27 and resumed on Day 37 at 300 mg/kg/day for all animals except one female, in which dosing resumed on Day 40. The pretomanid exposures are listed in Table 4.

Generally, monkeys at 450/300 mg/kg/day had clinical signs of hunched posture, inappetence, watery feces, decreased activity, and thin appearance over the course of the study. When the monkeys were given a dosing holiday and the dose level was lowered, as described above, the adverse effects were reversible. Male and females receiving 450/300 mg/kg/day lost body weight (−0.7% and −10.4% on average) over the course of the study compared to 22.3% and 11.7% mean body weight gain in control monkeys. Females administered 150 mg/kg/day also lost body weight (−1.7%) during the study, while males at 150 mg/kg/day had only a 6.1% gain. Body weights recovered for monkeys administered 450/300 mg/kg/day.

Clinical signs indicative of nervous system effects were observed in individual animals at 450/300 mg/kg/day. Specifically, ataxia was observed for two females at 450 mg/kg/day on Day 7, 27 or 28 of the study and one male administered 450/300 mg/kg/day on Days 62 and 70. The latter male also had convulsions on Days 62, 70, and 90 and a second male had convulsions on Day 58. One female was also observed with tremors on Day 31. The sex combined C_max_ and AUC_0–24 h_ on Day 90 for animals administered 450/300 mg/kg/day were 18.8 µg/mL and 295 µg·h/mL, respectively (Table 4).

During the dosing phase there were no ocular abnormalities detected. However, at the end of the 13-week recovery period, one male and one female administered 450/300 mg/kg/day had cortical axial posterior cataracts observed in each eye. No other lens changes were detected in any of the other monkeys.

There were no adverse effects on ECG, coagulation, urinalysis, or clinical chemistry parameters.

At necropsy, emaciation (body fat depletion) was observed in two females at 450/300 mg/kg/day and one male at 150 mg/kg/day, and small thymus was noted in one male at 450/300 mg/kg/day. Greater liver weights (males and females at 150 and 450/300 mg/kg/day) and lower thymus weight (males at 150 and 450/300 mg/kg/day and females at 50, 150, and 450/300 mg/kg/day) were observed. There were no similar findings in the recovery animals, indicating the reversibility of these changes.

The only drug-related microscopic findings were minimal to severe lymphoid depletion in both sexes at 150 and 450/300 mg/kg/day and diffuse, minimal, panlobular hepatocellular hypertrophy in all females at 450/300 mg/kg/day. At the recovery necropsy, microscopic lenticular lesions were noted in the male and female at 450/300 mg/kg/day that had cataracts, but evaluation of the nature of these lesions was complicated by fixation artifact noted microscopically in the same sections as well as in most study animals. Hepatocellular hypertrophy and thymic lymphoid depletion were reversible.

There were no marked sex differences in exposure to pretomanid. C_max_ increased less than dose-proportionally on Days 1, 7, and 90. Increases in AUC_0–24 h_ were generally dose proportional on Days 1 and 7. A less-than-dose-proportional increase in AUC_0–24 h_ was observed on Day 90 between the 150 and 300 mg/kg/day doses. Values for C_max_ and AUC_0–24 h_ generally increased from Day 1 to Day 90, indicating potential accumulation of pretomanid after repeat administration (Table 4).

Based on the results of this study, 450 mg/kg/day exceeded the MTD, as dosing had to be stopped after 4 weeks due to toxicity. After a dosing holiday followed by another 8 weeks of pretomanid at 300 mg/kg/day, emaciation, hepatocellular hypertrophy, and mild to severe thymic lymphoid depletion were present. Although no cataracts were observed at the end of dosing, after a 13-week recovery period two monkeys had bilateral cataracts; therefore, 300 mg/kg/day also was considered to exceed the MTD. The observation of cataracts during recovery led to the design of the 13-week investigative toxicity study in which no cataracts were observed (described below). At 150 mg/kg/day, females lost body weight and males only had a slight gain, and mild to moderate thymic lymphoid depletion was observed. These findings were conservatively considered to be adverse. No drug-related findings were observed at 50 mg/kg/day, and this was considered to be the 13-week NOAEL. After the last dose at the NOAEL, the sex-combined C_max_ and AUC_0–24 h_ were 8.0 µg/mL and 87 µg*h/mL, respectively.

### 3.4 13-week investigative toxicity study

Sexually mature male monkeys tolerated daily oral doses of pretomanid well at 50 mg/kg/day. At 150 and 300 mg/kg/day, monkeys were given a 7-day dosing holiday (Days 36–42) due to treatment-emergent toxicity and therefore were dosed for only 12 weeks. Additional dosing holidays occurred for four monkeys at 300 mg/kg/day beginning on Days 56, 59, or 75, so the total dose administrations for this group ranging from 47 to 84 days. Dose holidays or dose termination were required as the animals were not eating well and had lost substantial body weight (up to 1.9 kg or −25% vs controls).

Clinical signs indicating poor health were observed in the 150 and 300 mg/kg/day groups and consisted of hunched posture, ball position, and/or hypoactivity/lethargy. Decreased food consumption and body weight were noted in all animals in these groups. On Day 91 mean body weights in the 50, 150, and 300 mg/kg/day groups were 0.5, 1.4, and 1.2 kg less than that of the control monkeys, respectively. In general, all changes reversed during recovery.

The objective of this study was to evaluate effects of pretomanid on male reproductive system and lens in sexually mature cynomolgus monkeys. No pretomanid-related charges were observed during the monthly ophthalmic exams or during histopathology evaluation of the eyes. There was no pretomanid-related changes in testicular volume, serum testosterone, inhibin-B, LH, and FSH concentrations. Decreased sperm motility and total sperm count and increased abnormal-to-normal sperm ratio were noted in the 150 and 300 mg/kg/day groups. These changes in semen analysis parameters were considered to be secondary to declining physical condition (i.e., decreased food consumption and body weight loss) rather than direct effects of pretomanid on testes because the majority of these changes disappeared after recovery of physical condition and there were no histopathological correlates in the male genital organs.

No adverse clinical pathology findings were observed. There were also no pretomanid-related changes noted in gross pathology, organ weights, or histopathology, either at the end of the 13-week dosing phase or 20-week recovery phase of the study.

Exposure to pretomanid increased with increasing dose from 50 to 300 mg/kg/day. The increases in mean C_max_ and AUC_0–24 h_ were generally less than dose proportional. No accumulation of pretomanid was observed after multiple doses in monkeys (Table 4).

In conclusion, repeated daily administration of pretomanid at doses of 50 mg/kg for 13 weeks or at doses of 150 or 300 mg/kg for 12 weeks did not cause cataracts or testicular toxicity in sexually mature male monkeys.

### 3.5 39-week general toxicity study

Pretomanid was generally well tolerated. All animals except one survived until the end of the study. The exception was a male at 100 mg/kg/day that died on Day 90.

The male that died had frequent episodes of emesis during or immediately after dose administration between Days 9 and 71, and the technician noted on Day 10 that the monkey appeared to have a sensitive gag reflex. This monkey also had reduced appetite starting Day 81, decreased feces on Day 85, and tachypnea on Days 87–89. A blood sample taken Day 87 revealed mature neutrophilia (14,190/µL), monocytosis (1620/µL), eosinophilia (410/µL), hypoglycemia (42 mg/dL), low albumin concentration (2.8 g/dL), and high concentrations of globulin (3.7 g/dL) and fibrinogen (892 mg/dL). Veterinary evaluation revealed right-sided atelectasis due to a mass in the intrapleural space. Aspiration via percutaneous intercostal needle was unsuccessful due to consolidation of the mass, but analysis of the fluid indicated hyposegmented polymorphonucleur leukocytes. The veterinarian recommended surgery to remove the mass, but the animal died during surgery. The only noteworthy finding at necropsy was a 2-cm diameter thoracic mass full of gelatinous material. Microscopic examination revealed marked, suppurative, necrotizing pleuritis, widespread proliferation of alveolar macrophages containing foreign material, and a “suppurative granuloma” in the middle lobe of the lung. The cause of morbidity was considered to be aspiration pneumonia with pleuritis, most likely secondary to emesis and not directly related to pretomanid.

On Day 104 (after 15 weeks of dosing), a different male at 100 mg/kg/day experienced two convulsions following dose administration. The first convulsion occurred approximately 10 min postdose, after which the monkey was non-responsive, hypoactive, ataxic, and weak. The second convulsion occurred approximately 45 min postdose, after which the monkey was given diazepam (Valium®) intravenously twice. Dosing was suspended on Day 105 but resumed on Day 106. As per the attending veterinarian’s recommendation, the monkey was given 1 mg/kg of diazepam prior to dosing on Days 106–110. No further convulsions were seen during this time or for the duration of the study. Because convulsions occurred well before T_max_, did not recur in the affected monkey despite continued dosing, and were not seen in any other monkey at 100 mg/kg/day, the episode of convulsions was considered unlikely to be related to pretomanid; however, because the convulsions occurred at the high-dose level, an effect of pretomanid could not be ruled out.

No changes in ECG or ophthalmology parameters were attributed to treatment with pretomanid.

The only pretomanid-related clinical observation was reduced appetite, which occurred frequently and consistently in a dose-related manner in all groups. Reduced appetite was closely associated with a clear, dose-related decrease in body weight and decreased weight gain observed during treatment. Males and females in the 100 mg/kg/day group lost an average of 7.6% and 2.6% of prestudy body weight, respectively, during the first 13 weeks of the study. Most animals began to recover after 13 weeks of dosing. By the end of the recovery period (Week 52), males administered 100 mg/kg/day had gained 10.6% of their prestudy weights, but this gain was less than the gain in the control males (39%) during the same period. Females in this group had lost 5.3% of their prestudy body weight compared with a gain of 50% in the control recovery group females at the end of the recovery period.

Minor decreases in hemoglobin (males and females) and hematocrit (males) were observed throughout the study across all groups, coupled with increases in reticulocytes (absolute and % of red blood cells) in all pretomanid-treated females at Week 26; however, no meaningful or statistically significant changes in red blood cell (RBC) count were noted at any time during the study and no microscopic changes in bone marrow were noted in any dose group at either the main or recovery necropsy timepoints.

The incidence of dark yellow or yellow/red and/or turbid urine was higher in treated animals. This effect is possibly due to dehydration and resolved during the recovery period. Dark urine may also be a secondary effect of treatment with pretomanid, as metabolites of other nitroimidazoles have been shown to produce dark-colored urine [14].

There were no significant or clinically important effects of pretomanid on organ weights, although there was a dose-related increase in liver weight in males and the increased liver-to-body weight ratio was significant in high-dose males relative to control.

The only gross pathologic finding at necropsy related to pretomanid was visible thickening of the wall of the stomach and/or small intestine in almost all monkeys at all dose levels. In the small intestine, thickening was segmental. There were no histopathologic findings to account for the thickening, and no histopathologic findings in any tissue that were considered to be related to pretomanid. Gastrointestinal thickening showed evidence of resolving during the recovery period, as it was present only in the stomach of one 100 mg/kg/day male at the end of the recovery period.

After oral administration, the plasma T_max_ for pretomanid was between 2.5 and 4.0 h post-dose in the low-dose group and 3–6.5 h post-dose in the mid- and high-dose groups, suggesting pretomanid is slowly absorbed. Although peak drug levels increased with dose, these increases were generally less than dose proportional. No notable differences between males and females or between single and repeated administration were observed. AUC_inf_ increased with dose, but the increases were not dose proportional with the exception of females at 100 mg/kg/day, which increased greater than in proportion to dose on Days 15, 92, and 260 (Table 4).

In summary, oral administration of pretomanid to cynomolgus monkeys for 39 weeks at doses up to 100 mg/kg/day was generally well tolerated. Loss of appetite and corresponding weight loss were observed during the first 3 months of the study, although animals gradually acclimated to the drug, with corresponding improvements in food consumption and body weight. The only gross pathologic finding related to pretomanid was thickening of the wall of the stomach and/or small intestine in most monkeys at all dose levels. Thickening showed evidence of resolving during the recovery period, as it was present only in the stomach of one high-dose male at the end of the recovery period. The macroscopic thickening was not associated with any inlife or postmortem evidence of an effect on gastrointestinal tract function or general health (i.e. emesis, loose stools, diarrhea), and therefore was not considered adverse. The lower body weight gain in females ≥ 50 mg/kg/day was considered adverse, and therefore the 25 mg/kg/day was considered the NOAEL for females. Weight loss in two males at 100 mg/kg/day was adverse and therefore 50 mg/kg/day was considered the NOAEL in males. On Day 260, the mean C_max_ and AUC_last_ (essentially AUC_0–24 h_) values for females administered 25 mg/kg/day were 2.6 µg/mL and 17.9 µg*h/mL, respectively. On Day 260, the mean C_max_ and AUC_last_ (essentially AUC_0–24 h_) values were 5.2 µg/mL and 55.4 µg*h/mL, respectively.

## 4 Discussion

Pretomanid toxicity in monkeys was characterized in a series of repeat-dose toxicity studies of increasing duration. In a 2-week study, pretomanid was administered up to 1000 mg/kg/day which produced exposures greater than 10-fold higher than anticipated human exposures, allowing characterization of dose-limiting toxicity (Table 2, Table 3). NOAELs were identified in all toxicity studies. At the NOAELs, average pretomanid exposure equaled or exceeded human exposure at the approved pretomanid dose (200 mg/day, mean AUC_0–24 h_ 50.9 µg*h/mL) with the exception of female monkeys in the 39-week study (mean AUC_0–24 h_ 20 µg*h/mL) which was determined by lower weight gains for the mid and high dose levels and is a clinically monitorable finding. As study duration increased, no new toxicities were observed, but the NOAEL decreased (Table 3).

Based on toxicity studies in monkeys, the heart (decreased heart rate and QT prolongation), eyes (cataracts), central nervous system (ataxia, tremor, convulsion), and liver (hepatocellular hypertrophy without elevations in alanine aminotransferase [ALT] or aspartate aminotransferase [AST] activities) were identified as potential target organs for pretomanid toxicity in human subjects, as outlined in Table 2 [15]. The concordance between the findings in monkeys and from clinical studies assessing the safety of pretomanid as a monotherapy will be briefly discussed.

### 4.1 Decreased heart rate and QT prolongation

In the 2-week study, monkeys given pretomanid had decreased heart rate and increased QT interval duration at ≥ 450 mg/kg/day but not at ≤ 150 mg/kg/day. Because of limitations described in the results section in the 2-week study, definitive conclusions on the effects of pretomanid on QT interval, independent of heart rate, could not be made. No effects on heart rate or QT interval duration were seen in monkeys given pretomanid up to 300 mg/kg/day for 12 weeks or up to 100 mg/kg/day for 39 weeks.

A standalone safety pharmacology package was conducted with pretomanid to definitively evaluate effects of the QT interval (data unpublished). In vitro, pretomanid inhibited current mediated by human Ether-a-go-go Related Gene (hERG) potassium channels at a concentration approximately 2-fold above the anticipated human exposure (C_max_) at the approved pretomanid dose, indicating pretomanid had the potential to delay ventricular repolarization. In vivo cardiovascular studies in surgically instrumented monkeys monitored continuously demonstrated evidence of QT prolongation after a single dose of pretomanid at 150 mg/kg. Pretomanid was also tested in combination with bedaquiline in dogs and moxifloxacin in monkeys, and there was no evidence of an interactive effect on QT prolongation in these studies (unpublished data).

The slight QT prolongation observed in monkeys has not been observed as clinically meaningful QT prolongation during clinical trials. In Phase I studies in healthy patients, no cardiovascular-related effects on 2-lead cardiac profiles or 12-lead ECG parameters, including heart rate and QT interval, were noted after single doses of pretomanid up to 1500 mg or seven repeat doses up to 600 mg [16]. In a randomized, double-blind, placebo- and positive controlled, crossover, thorough QT study of pretomanid, single doses of pretomanid up to 1000 mg caused no QTc prolongation of clinical concern in healthy subjects [17]. Pretomanid monotherapy during two early bactericidal activity (EBA) studies up to 1200 mg for 14 days was well tolerated with no clinically significant QTc changes [18], [19].

### 4.2 Cataracts

No pretomanid-related cataracts were present at the end of dosing in any of the toxicity studies in monkeys, but were identified at the end of the treatment-free period in the 13-week general toxicity study in 2 of 4 monkeys given pretomanid at 450 mg/kg/day for 4 weeks and then 300 mg/kg/day for 8 weeks. These findings were further investigated in a follow-up 13-week investigative toxicity study designed to investigate ocular changes and a 39-week general toxicity study. In the latter studies no pretomanid-related cataracts or ocular abnormalities were observed.

Specifically, in the subsequent 13-week investigative toxicity study, cataracts were not seen in 8 male monkeys given pretomanid at 300 mg/kg/day for up to 12 weeks, nor were cataracts seen in the 4 of them that were maintained for an additional 20-week recovery period. Frequent eye exams were conducted, but no cataracts were observed. Additionally, cataracts were not observed in monkeys given pretomanid at up to 100 mg/kg/day pretomanid for 39 weeks.

The 39-week NOEL for cataracts in monkeys was 100 mg/kg/day, which produced average C_max_ and AUC_0–24 h_ values of 10.0 μg/mL and 124 h* μg/mL, respectively. These values are more than twice the exposure in patients at the approved pretomanid dose (Table 3).

In clinical trials, subjects were monitored for cataracts and lens disorders and slit lamp examinations were conducted when pretomanid was administered for more than 14 days. No clinically meaningful effect on cataract formation or lens disorders were identified in Phase 2 pretomanid-alone groups in drug-sensitive TB patients. When examining for lens disorders, normal variation in rater variability and/or age-related changes were noted, with no clinically meaningful effect of pretomanid on the potential for cataract formation in any clinical study.

### 4.3 Nervous system/convulsions

In monkeys, nervous system-related clinical signs included ataxia and convulsions followed by lateral recumbency and ataxia, or front limb tremors at a dose of 1000 mg/kg/day given for two weeks. In longer repeat-dose studies in which monkeys were given ≥ 100 mg/kg/day pretomanid, ataxia and convulsions were rare but observed in 2 studies. Nervous system-related clinical signs were not seen in monkeys dosed for 39 weeks at ≤ 50 mg/kg/day, and so this was considered to be the 39-week NOEL for nervous system effects. After 39 weeks of administration at the NOEL, mean C_max_ and AUC_0–24 h_ values were greater in males than in females and were approximately 5.2 µg/mL and 55.4 h* µg/mL, respectively. These values are higher than (C_max_) or similar to (AUC_0–24 h_) the predicted exposures in patients at the approved pretomanid dose of 200 mg/day (Table 3).

In Phase I and II pretomanid-alone clinical studies, no convulsions were reported.

### 4.4 Hepatocellular hypertrophy

Hepatocellular hypertrophy was observed in monkeys given oral doses of pretomanid at ≥ 100 mg/kg/day and was considered an adaptive response associated with increased metabolism. In the 39-week study, there was a dose-related increase in liver weights in males at all dose levels, but this was not associated with histopathologic findings. Pretomanid plasma exposures where increased liver weights were observed in monkeys was at least 1.5-fold higher than the anticipated human exposure. There were no elevations of ALT or AST in the monkey studies.

In Phase 2 pretomanid-alone pooling groups in the pretomanid program, increases in ALT and AST activities > 3 and ≤ 5 times the upper limit of normal were observed in 1 (0.8%) and 2 subjects (1.6%), respectively, and increases in these parameters > 5 and ≤ 8 times the upper limit of normal were observed in 1 (0.8%) and 0 subjects (0%), respectively. No pretomanid subject had an increase in ALT or AST > 8 times upper limit of normal or an increase in total bilirubin above the upper limit of normal. No subject in the Phase 2 pretomanid-alone pooling group met laboratory criteria for a potential Hy’s law case.

### 4.5 Pretomanid safety in BPaL regimen

Nonclinical combination toxicology studies were not conducted with the BPaL regimen based on considerations of the toxicologic profile of the individual drugs in the regimen and the FDA agreed with this strategy.

The Nix-TB trial, which was the pivotal clinical trial for BPaL regimen approval, identified most dose-limiting toxicities related to linezolid and not pretomanid. The most common adverse events reported from the Nix-TB trial were associated with linezolid treatment and included peripheral neuropathy in 81% of patients and in the majority of cases the symptoms were mild to moderate [7], followed by myelosuppression (48%), and anemia (37%). Most patients had linezolid treatment interruption or reduction in linezolid dose. Other common side effects (>15%) noted with the BPaL regimen included acne (39%), nausea (37%), vomiting (34%), musculoskeletal pain (29%), headache (28%), dyspepsia (24%), decreased appetite (22%), rash (21%), pruritus (20%), abdominal pain (19%), pleuritic pain (17%), increased gamma glutyl transpeptidase (17%), and lower respiratory tract infection (15%)[15]. In patients that had the BPaL regimen interrupted for hepatic adverse events, all were able to resume and complete full treatment [7].

## 5 Conclusions

The program of repeat-dose toxicity studies in monkeys with pretomanid thoroughly characterized its toxicity profile in this species and successfully supported clinical development and eventual approval of pretomanid by the United States FDA and in more than 10 other countries globally as part of the BPaL three-drug, six-month, all-oral regimen for treatment of pulmonary XDR or complicated forms of MDR-TB. There was no evidence from clinical safety data that any of the four target organ toxicities identified in monkeys — decreased heart rate/increased QT interval duration, cataracts, ataxia/tremor/convulsion, or hepatocellular hypertrophy — occurred in human subjects during clinical development. This does not mean that monkeys were not good predictors for humans. Instead, the absence of these effects in human subjects most likely reflects the fact that systemic exposure to pretomanid at the dose levels evaluated clinically was below the threshold for the effects seen in monkeys.

## Funding

The project was supported by TB Alliance with funding from Australia’s Department of Foreign Affairs and Trade, Bill & Melinda Gates Foundation (Grant Number: OPP1129600), Germany’s Federal Ministry of Education and Research through KfW, Irish Aid, National Institute of Allergy and Infectious Disease, Netherlands Ministry of Foreign Affairs, United Kingdom Department for International Development, and the United States Agency for International Development.

## CRediT authorship contribution statement

**Rebecca Bruning-Barry:** Writing – original draft, Writing – review & editing, Visualization, Project administration. **Jeffrey L. Ambroso:** Writing – original draft, Writing – review & editing, Project administration. **John Dillberger:** Writing – original draft, Writing – review & editing, Project administration. **Tian J. Yang:** Writing – original draft, Writing – review & editing, Supervision, Project administration.

## Declaration of Competing Interest

The authors declare the following financial interests/personal relationships which may be considered as potential competing interests: Tian Yang was an employee of the TB Alliance (The Global Alliance for TB Drug Development), a non-profit organization dedicated to the discovery and development of improved TB therapeutics. The TB Alliance is funded by governments and foundations, through which this research was supported. Rebecca Bruning-Barry and Jeffrey Ambroso are employees of RTI International, an independent, non-profit scientific research and development institute, and consult on TB Alliance projects. John Dillberger is an independent consultant on TB Alliance projects.

## References

[1] Stover C.K., Warrener P., VanDevanter D.R., Sherman D.R., Arain T.M., Langhorne M.H., Anderson S.W., Towell J.A., Yuan Y., McMurray D.N., Kreiswirth B.N., Barry C.E., Baker W.R. (2000). A small-molecule nitroimidazopyran drug candidate for the treatment of tuberculosis. Nature.

[2] Singh R., Manjunatha U., Boshoff H.I., Ha Y.H., Niyomrattanakit P., Ledwidge R., Dowd C.S., Lee I.Y., Kim P., Zhang L., Kang S., Keller T.H., Jiricek J., Barry C.E. (2008). PA-824 kills nonreplicating Mycobacterium tuberculosis by intracellular NO release. Science.

[3] Manjunatha U., Boshoff H.I., Barry C.E. (2009). The mechanism of action of PA-824: novel insights from transcriptional profiling. Commun. Integr. Biol..

[4] World Health Organization (WHO). 2021. Meeting report of the WHO expert consultation on the definition of extensively drug-resistant tuberculosis, 27–29 October 2020. WHO, Geneva.

[5] World Health Organization (WHO) (2020).

[6] World Health Organization (WHO) (2018).

[7] Conradie F., Diacon A.H., Ngubane N., Howell P., Everitt D., Crook A.M., Mendel C.M., Egizi E., Moreira J., Timm J., McHugh T.D., Wills G.H., Bateson A., Hunt R., Van Niekerk C., Li M., Olugbosi M., Spigelman M. (2020). Treatment of highly drug-resistant pulmonary tuberculosis. New Engl. J. Med..

[8] Black T.A., Buchwald U.K. (2021). The pipeline of new molecules and regimens against drug-resistant tuberculosis. J. Clin. Tube Other Mycobact. Dis..

[9] J. Trutter, R. Dalefield, 7-Day Gavage Study with PA-824 in Cynomolgus Monkeys Covance Lab. Inc. 2004.

[10] J. Trutter, R. Dalefield, 2-Week Nasogastric Gavage Toxicity and Toxicokinetic Study with PA-824 in Cynomolgus Monkeys with a Minimum 2-Week Recovery Period Covance Lab. Inc. 2005.

[11] E. Goldenthal, PA-824: A 3-Month Oral Toxicity Study in Cynomolgus Monkeys with a 3-Month Recovery MPI Res. Inc. 2008.

[12] S. Oneda ,PA-824: A 13-Week Study to Evaluate Reproductive Function and Lenticular Lesions in Male Cynomolgus Monkeys SNBL USA, Ltd 2009.

[13] Gahagen J. (2015). 39-week repeat-dose toxicology and toxicokinetic study with PA-824 in mature cynomolgus macaques with a 12-week recovery period. SRI Int..

[14] Bruce T.A. (1971). Dark urine related to metronidazole therapy. JAMA.

[15] Mylan Pretomanid [Label. ], Vol. 212862Orig1 2019 s000.

[16] Ginsberg A.M., Laurenzi M.W., Rouse D.J., Whitney K.D., Spigelman M.K. (2009). Safety, tolerability, and pharmacokinetics of PA-824 in healthy subjects. Antimicrob. Agents Chemother..

[17] Li M., Saviolakis G.A., El-Amin W., Makhene M.K., Osborn B., Nedelman J., Yang T.J., Everitt D. (2021). Phase 1 study of the effects of the tuberculosis treatment pretomanid, alone and in combination with moxifloxacin, on the QTc interval in healthy volunteers. Clin. Pharm. Drug Dev..

[18] Diacon A.H., Dawson R., du Bois J., Narunsky K., Venter A., Donald P.R., van Niekerk C., Erondu N., Ginsberg A.M., Becker P., Spigelman M.K. (2012). Phase II dose-ranging trial of the early bactericidal activity of PA-824. Antimicrob. Agents Chemother..

[19] Diacon A.H., Dawson R., Hanekom M., Narunsky K., Maritz S.J., Venter A., Donald P.R., van Niekerk C., Whitney K., Rouse D.J., Laurenzi M.W., Ginsberg A.M., Spigelman M.K. (2010). Early bactericidal activity and pharmacokinetics of PA-824 in smear-positive tuberculosis patients. Antimicrob. Agents Chemother..

